# Hybrid glenoid component migration after total shoulder arthroplasty: a cohort study using radiostereometric analysis with 2 years’ follow-up

**DOI:** 10.2340/17453674.2025.44953

**Published:** 2025-11-13

**Authors:** Adriano A CECCOTTI, Mikkel TØTTRUP, Mogens LAURSEN, Hans-Christen HUSUM, Steen L JENSEN

**Affiliations:** 1 Orthopedic Department, Interdisciplinary Orthopedics, Aalborg University Hospital, Aalborg; 2 Orthopedic Department, Aarhus University Hospital, Aarhus; 3 Department of Radiology, Randers Regional Hospital, Randers, Denmark

## Abstract

**Background and purpose:**

Anatomic total shoulder arthroplasty (aTSA) is an effective treatment for glenohumeral osteoarthritis, but loosening of the glenoid component is a common cause for revision. Our primary aim was to analyze migration of a hybrid glenoid component in aTSA using marker-based radiostereometric analysis (RSA). Second, we aimed to compare early migration with later revision.

**Methods:**

Patients with primary glenohumeral osteoarthritis treated with a hybrid glenoid aTSA had tantalum markers inserted in the scapular bone and the glenoid component from 2017 to 2020. We used hybrid glenoid fixation techniques combining cementation with bone in-growth. Patients were followed with RSA radiographs for 24 months. Migration analyses included translation, rotation, and MTPM (maximum total point of motion). Data regarding revisions was retrieved from the Danish Shoulder Arthroplasty Register.

**Results:**

72 patients were included (mean age 69 [SD 8] years, 41 females, 37 left shoulders). The mean MTPM at 24 months was 0.97 mm (95% confidence interval [CI] 0.85–1.1), but migration occurred mainly within the first 6 months (MTPM 0.88 mm, CI 0.78–0.98). The predominant movement was valgus rotation. 2 cases were revised and both had positive cultures. 1 of these had major migration and was found to be loose at revision.

**Conclusion:**

The hybrid glenoid components migrated mainly within the first 6 months (initial seating). Thereafter the components reached a plateau phase and stabilized. Due to the few observations and the relatively short study period, association between early migration and later revision could not be evaluated.

Anatomic total shoulder arthroplasty (aTSA) is recommended for the treatment of glenohumeral osteoarthritis [1]. It is associated with pain relief and improved range of motion [2,3].

Loosening of the glenoid component is of concern, and high rates of radiolucency lines have been reported, suggesting component loosening [4,5]. Beside instability, rotator cuff insufficiency, and infection, loosening of the glenoid remains a common indication for revision of aTSA [6,7]. All-polyethylene components with pure cemented fixation have so far demonstrated the best revision-free survival, but other designs have emerged to improve the longevity of the glenoid implants [8]. Hybrid glenoid components have been developed with the idea to combine initial stability by cementation and long-term stability by bone in-growth [9-11].

Radiostereometric analysis (RSA) is an accurate method of determining the migration of orthopedic implants [12,13]. Progressive component micromotion detected by RSA was associated with later clinical and radiographic revision for knee and hip arthroplasties [14-16]. Only a few RSA studies have been conducted for aTSA, but it has been feasible to employ the technique to investigate glenoid component migration [17-20]. Although hybrid glenoid components have been available for years, only 1 study has investigated micromotions with RSA [21]. This component was an all-polyethylene with a modified central peg with flutes designed for uncemented fixation, but not for osseointegration.

At our institution, we used an aTSA with a porous-coated central titanium post on the glenoid as our standard arthroplasty for glenohumeral osteoarthritis for more than 10 years. During the period of this study, tantalum beads were inserted routinely, and patients were scheduled for regular radiographic follow-up. The primary aim of our study was to analyze the 2-year migration of a hybrid glenoid component in aTSA using marker-based RSA technique. Our secondary aim was to investigate the association between early migration and later revision.

## Method

### Study design

This was a prospective marker-based RSA implant safety study performed at the Aalborg University Hospital during the period from January 2017 to February 2022. The results are reported according to the STROBE guideline for observational studies and according to current RSA guidelines [22,23].

### Study group

An aTSA with a glenoid component designed for hybrid fixation was the standard arthroplasty used for osteoarthritis in our department. It is designed with 3 peripheral pegs for cementation and a central porous-coated titanium post for bone ingrowth (Comprehensive, Zimmer Biomet, Inc, Warsaw, IN, USA). During the period of this study, tantalum beads were inserted whenever possible as part of the standard procedure. Only patients with primary glenohumeral osteoarthritis were included. The surgeon determined rotator cuff status preoperatively by ultrasonography, and only patients with intact rotator cuff were scheduled for aTSA.

The latest preoperative radiographs were used to grade the severity of osteoarthritis according to Samilson and Prieto [24]. The modified Walch classification was used to assess glenoid morphology with preoperative CT scans reformatted according to the anatomic axes of the scapula [25]. Friedmann’s line was used to measure the glenoid erosion and humeral subluxation [26]. Both the Samilson–Prieto grade and the modified Walch classification were performed independently by 2 authors, each with at least 6 years of experience in the orthopedic field. In the case of disagreement, final agreement was achieved by discussion.

### Marker insertion technique

**Figure 1:**
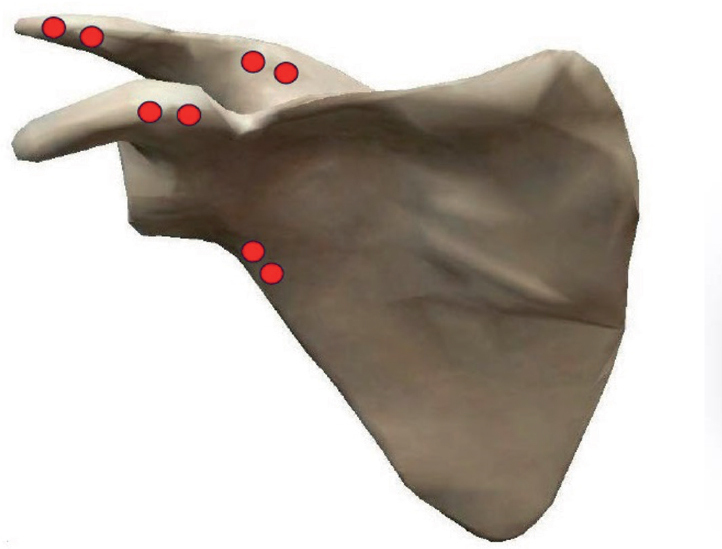
Right scapula showing the positions of the tantalum markers (red dots). Visible Body Suite: Human Anatomy Atlas 2020 Retrieved from www.visiblebody.com

**Figure 2:**
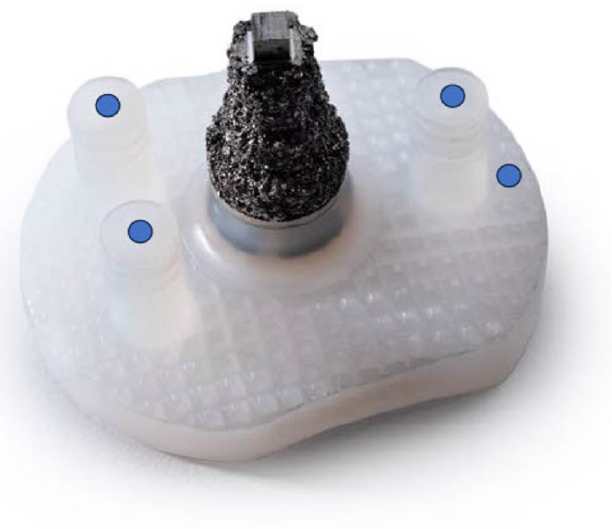
Hybrid glenoid component showing the position of the tantalum markers (blue dots).

The arthroplasty procedures were performed according to the manufacturer’s recommendations by surgeons specialized in shoulder surgery; during the study period 6 surgeons were involved in the surgeries. During the surgical procedures, 1 mm tantalum markers were inserted at 4 positions in the scapular bone (spine, acromion, coracoid process, lateral margin; Figure 1). Due to the risk of losing markers in the soft tissue, 2 markers were inserted at each position approximately 1 cm apart. With the use of a guide canula, a hole was drilled in the bone with a 1 mm Kirschner wire followed by insertion of the marker. Glenoid component markers were inserted at 4 positions (one in each peg and one at the top; Figure 2). The markers were pushed into 3 mm deep holes made with a 1 mm drill bit. A bead gun (Kulkanon, Wennbergs Finmek, Sweden) was used for the insertion of the markers in the bone and the implant. Markers were distributed in approximately the same pattern and in a manner creating the largest possible 3D reconstruction, aiming at a low condition number. The tip of the central post was used as a marker during analysis by generating an elementary geometrical shape.

### Radiographs

**Figure 3:**
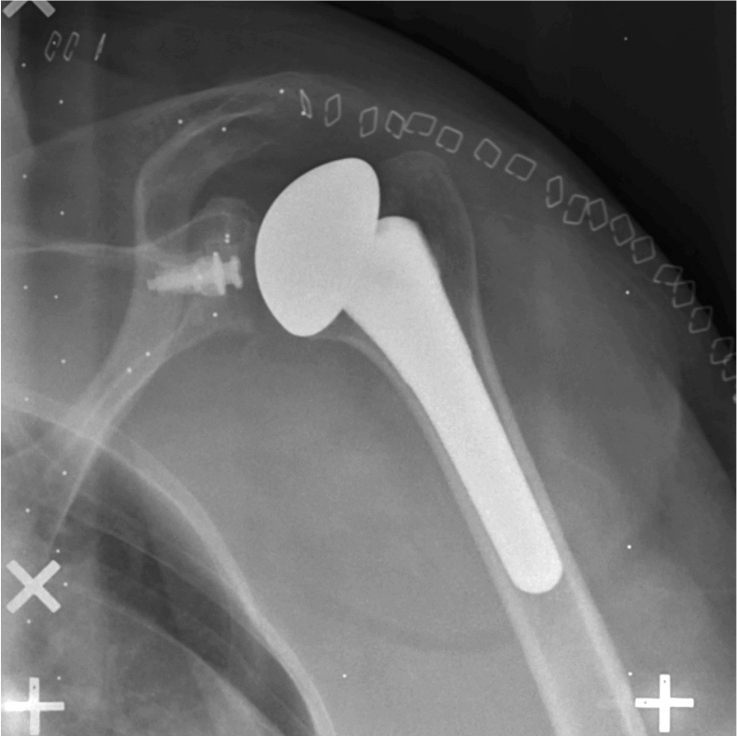
Left anatomic total shoulder arthroplasty with 1 mm tantalum markers.

According to the guidelines for RSA [22], RSA radiographs were obtained using a uniplanar radiographic arrangement (Figure 3). X-ray tubes were positioned over a uniplanar calibration cage, Calibration Cage No. 43 (RSA Biomedical, Umeå, Sweden). The patient was positioned supine and rotated approximately 45° with the scapula of the operated side lying flat against the table. RSA radiographs were taken on the first postoperative day to serve as reference. These radiographs were evaluated and if found insufficient, the patient was excluded. The remainder were scheduled for follow-up radiographs at regular intervals until 24 months (6 weeks, 3 months, 6 months, 12 months, 24 months) postoperatively.

### RSA

RSA was performed using Model-based RSA 4.1 software (RSAcore, Department of Orthopedic Surgery, Leiden University Medical Center, the Netherlands). The migration of the glenoid relative to the scapula was expressed as translation (mm), rotation (°), and maximum total point motion (MTPM; mm) around the 3 axes (x, y, and z).

Anatomically, translations about the orthogonal axes within the coordinate system were expressed as lateral to medial translation (transverse or x-axis), inferior to superior translation (longitudinal or y-axis), and posterior to anterior translation (sagittal or z-axis). Positive rotations about the orthogonal axes were expressed as anterior for the transverse or x-axis, inward for the longitudinal or y-axis, and varus for the sagittal or z-axis. The MTPM was calculated as the vectorial length of the translation of the point in the rigid body that had the greatest motion [22]. Migration results of the left shoulder were transformed to a right-side shoulder [27].

In terms of RSA analysis, the condition number (CN) reflects the distribution of the markers. Cases with CN > 120 or with a mean error for rigid body fitting > 0.35 were excluded. The RSA analysis needed at least the same 3 markers throughout the various timepoints to follow the 3D geometrical shape, which is why RSA analyses with matched markers (MM) < 3 were excluded [22].

### RSA clinical precision

For the purpose of establishing the precision of the RSA measurements, double examinations were performed at 12 months’ follow-up with complete repositioning of the patient to estimate the clinical precision (accuracy of zero motion) of the study’s setup [22,28].

### RSA clinical precision

Of the 60 cases that contributed with 12 months’ RSA radiographs, only 53 had double radiographs that were acceptable (Table 1). Of the 7 missing, 1 had erroneously only 1 set of radiographs taken. The remaining had 1 of the 2 sets discarded due to less than 3 matching markers (1 case), CN greater than 120 (2 cases), or overlay of the humeral head (3 cases).

**Table 1:** Results from double examinations at 12 months (N = 53)

Item	Mean difference (SD)	Range	Precision
Translation, mm			
X	0.09 (0.10)	0.002–0.43	0.20
Y	0.05 (0.06)	0.000–0.34	0.12
Z	0.12 (0.11)	0.001–0.44	0.22
Rotation, °			
X	0.37 (0.41)	0.001–2.42	0.80
Y	0.86 (0.84)	0.011–3.87	1.65
Z	0.33 (0.31)	0.010–1.67	0.61
MTPM, mm	0.15 (0.16)	0.002–0.62	0.31

Precision = 1.96 x SD of the difference

### Revisions

To determine any association between component migration and later revision, data regarding revision for the cohort was retrieved from the Danish Shoulder Arthroplasty Registry by September 1, 2022. The completeness of registration to the registry has been above 92% since 2017 [29]. Revision was defined as the addition, exchange, or removal of any component in an existing arthroplasty. The revision status of each case was cross-checked with the electronic patient records.

### Statistics

The results of the RSA (translation, rotation, MTPM) were calculated as means, standard deviation (SD), and 95% confidence interval (CI). Differences in translation, rotation, or MTPM during follow-up were compared using repeated measures ANOVA. The precision of RSA was calculated as 1.96 x SD of the mean paired difference of the double examinations [27], where the mean represents the systematical error of the method [30]. Normal distribution of data was confirmed using quantile–quantile plots and a significance level of 5% was used. Statistical data analysis was done using STATA 17 (Stata Statistical Software: Release 17. 2021. StataCorp LLC, College Station, TX, USA).

### Ethics, data sharing plan, use of AI, funding, and disclosures

Permissions to obtain and retrieve registry data and study patient records including diagnostics imaging were given by the North Region Denmark (ID: 2021-035119). According to the Danish National Committee on Health Research Ethics, the current study did not require their approval. Insertion of tantalum beads and regular radiographic follow-up was part of the standard procedure for our aTSA. The patients were given this information together with other relevant information concerning the procedure and gave their informed consent before surgery. Data sharing is currently not possible but is in progress. Please contact the corresponding author for information about the process. No AI tools has been used in the study. “Interdisciplinary Orthopaedics” at Aalborg University and Zimmer Biomet has co-financed the study. The authors report no conflicts of interests. Complete disclosure of interest forms according to ICMJE are available on the article page, doi: 10.2340/17453674.2025.44953

## Results

**Figure 4:**
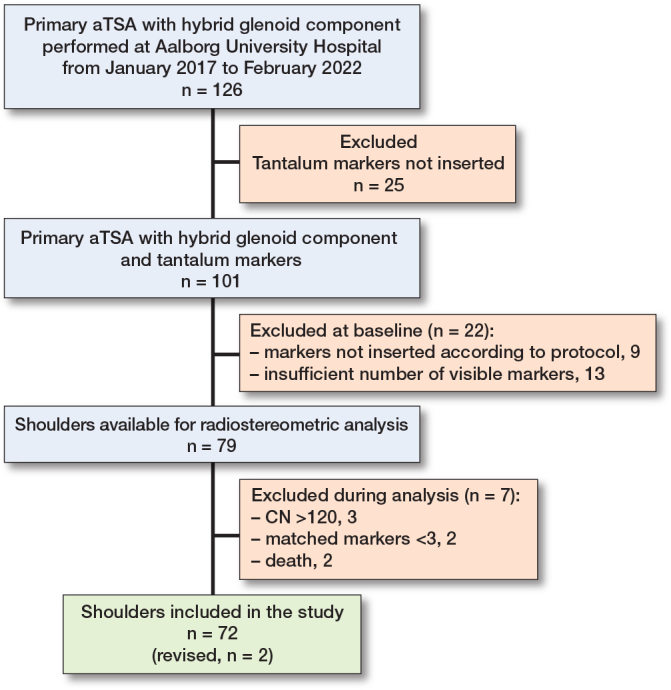
Flowchart showing inclusion and exclusion.

126 cases with primary osteoarthritis were treated with an aTSA during the study period, all using the hybrid glenoid. Tantalum beads were not inserted in 25 cases, either because the surgeon was unfamiliar with the technique of marker insertion or there was a lack of equipment such as bead-guns. Of the 101 eligible cases, 22 were excluded during evaluation and analysis of the baseline postoperative radiographs: 9 cases because the markers had not been inserted correctly, 13 cases due to insufficient number of visible markers (e.g., reference bone markers or protheses markers were overlaid by the humeral head, leaving less than 3 possibly matched markers), 3 cases due to high CN, and 2 because there were less than 3 matching markers throughout all RSA radiographs (Figure 4). None was excluded due to high error for rigid body fitting. 2 cases were dismissed due to death and 2 cases were revised.

Thus, 72 patients were included with basic characteristics as shown in Table 1. The Samilson–Prieto classification was not possible in 1 case because the preoperative radiographs could not be retrieved. The modified Walch classification was possible in 65 cases; the remaining had no preoperative CT scans. Most patients (72%) had Walch type B with posterior erosion (Table 2).

**Table 2:** Preoperative baseline characteristics. Values are count unless otherwise specified

Sex, female / male	40 / 32
Age, median (range)	69 (47–87)
Left / right shoulder	36 / 36
BMI, mean (SD), n = 70	30 (5)
ASA score	
1	13
2	40
3	14
Missing	5
Samilson–Prieto	
No osteophyte	1
Mild (< 3 mm)	2
Moderate (3–7 mm)	4
Severe (> 7 mm)	64
Missing	4
Modified Walch classification	
A1	10
A2	7
B1	21
B2	18
B3	8
D	1
Missing	7

ASA = American Society of Anesthesiologists physical status

### Migration

Table 3 summarizes migrations along the 3 axes and the MTPM. Dropouts occurred at each time point, either from missed patient appointments or from technical errors during analysis.

**Table 3:** Translation, rotation, and MTPM at each timepoint (N = 72)

Follow up	6 weeks	3 months	6 months	12 months	24 months
Available, n	66	67	62	60	63
Missing, n					
No show	3	2	2	3	3
Poor radiographs	2	–	–	–	–
CN > 120	–	3	6	5	4
MM < 3	1	–	2	4	2
Translation, mm, mean (SD) [range]					
X (+ medial)	0.09 (0.18)	0.13 (0.23)	0.13 (0.31)	0.12 (0.29)	0.18 (0.30)
	[–0.42 to 0.64]	[–0.36 to 0.9]	[–1.20 to 1.02]	[–0.92 to 0.96]	[–0.45 to 1.07]
Y (+ proximal)	0.03 (0.14)	0.01 (0.18)	–0.02 (0.19)	–0.02 (0.21	–0.02 (0.19
	[–0.48 to 0.48]	[–0.95 to 0.36]	[–0.64 to 0.34]	[–0.77 to 0.37]	[–0.45 to 0.45]
Z (+ anterior)	–0.01 (0.18)	0.01 (0.25)	0.00 (0.27)	0.00 (0.27	0.07 (0.29
	[–0.45 to 0.41]	[–0.66 to 0.69]	[–0.81 to 0.64]	[–0.89 to 0.67]	[–0.78 to 1.46]
Rotation °, mean (SD) [range]					
X (+ anterior)	–0.12 (0.55)	–0.10 (0.74)	–0.16 (0.96)	–0.24 (0.98)	–0.10 (1.00)
	[–1.39 to 1.31]	[–1.84 to 1.61]	[–2.81 to 2.55]	[–2.55 to 2.62]	[–2.64 to 1.81]
Y (+ inward)	0.00 (1.47)	–0.08 (1.62)	–0.19 (2.04)	–0.18 (1.79	–0.12 (2.08)
	[–3.06 to 4.7]	[–5.25 to 3.99]	[–4.33 to 6.38]	[–4.27 to 5.36]	[–5.45 to 4.5]
Z (+ varus)	–0.45 (0.86)	–0.88 (1.29)	–1.18 (1.87)	–1.54 (2.15)	–1.70 (2.04)
	[–3.58 to 1.57]	[–5.99 to 1.18]	[–5.66 to 6.98]	[–7.32 to 8.23]	[–9.36 to 1.04]
MTPM, mm, mean (SD) [CI]					
	0.61 (0.04)	0.73 (0.06)	0.88 (0.08)	0.90 (0.08)	0.97 (0.10)
	[0.54 to 0.69]	[0.64 to 0.81]	[0.78 to 0.98]	[0.79 to 1.02]	[0.85 to 1.10]

CN = condition number; MM = matched markers; MTPM = maximum total point of motion.

**Figure 5:**
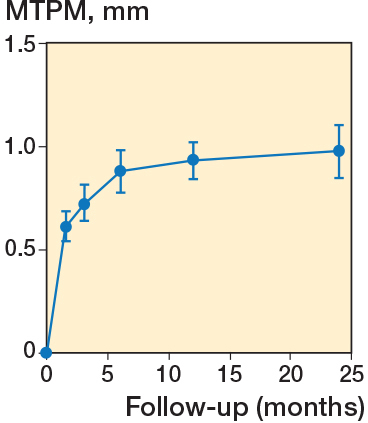
Mean maximum total point of motion (MTPM) at each timepoint. Error bars indicate 95% confidence intervals.

There was significant migration from surgery to 24 months’ follow-up as expressed by the mean MTPM, which reached 0.97 mm (CI 0.85–1.1; P < 0.001) at 24 months (see Table 2). Most of the migration occurred during the first 6 months after which there was only a slight, non-significant, increase in mean MTPM (Figure 5).

**Figure 6:**
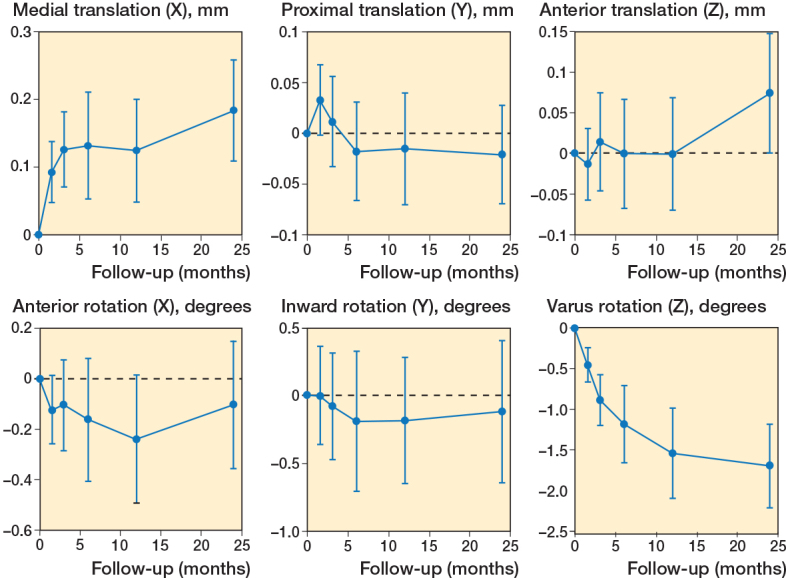
Translations (mm) and rotations (°) during follow-up. Means and 95% confidence intervals are shown for each axis. Note that all rotations are negative, i.e., posterior (X), outward (Y), and valgus (Z).

The migration pattern consisted primarily of a negative rotation about the z-axis, valgus rotation, with a mean rotation of –1.7° after 24 months, and a medial translation along the x-axis (medialization) with a mean translation of 0.18 mm after 24 months (Figure 6). Only the rotation about the z-axis was statistically significant (P <0.001).

### Revisions

The mean observation period regarding revisions was 49 months (range 34–68). We found 2 cases of revision, performed 23 and 27 months after index surgery, respectively. Both cases were revised to a reverse total shoulder arthroplasty and had perioperative samples that were positive for *Cutibacterium acnes.* In both cases, the last RSA radiographs were taken at 12 months. In the first case, MTPM was 1.88 mm and 1.80 after 6 and 12 months, respectively. The positive major rotation was about the z-axis, varus, reaching 8.2° at 12 months. This glenoid component was described as loose during revision surgery. In the second case, MTPM was 1.27 mm after 6 months, but only 0.41 mm after 12 months. Accordingly, there was an inward rotation (y-axis) of 4.1° and a varus rotation of 1.4° after 6 months, which was –1.17° and 0.16° after 12 months, respectively. Although the measurements suggest some loosening, the component was not described as loose during revision surgery (see Tables 4 and 5 for migration data in the revised cases in Supplementary data).

## Discussion

This is the first RSA study investigating early migration of a hybrid glenoid component with a central porous metal post meant for biological fixation. Our primary aim was to analyze migration of a hybrid glenoid component in aTSA using marker-based RSA.

We found a statistically significant migration in terms of MTPM mainly within the first 6 months; thereafter the glenoid component reached a plateau phase, indicating stability. This finding may indicate that seating is taking place during the first 6 months, until final stability. This may be explained by bone in-growth to the central post but the same pattern has been found for some all-polyethylene components [19].

Both revised cases in this series displayed larger migration and an aberrant migration pattern compared with the unrevised components. Although this suggests early migration as a risk factor for later revision, we believe longer follow-up with more events is needed to determine a more certain association.

Nuttall et al., however, investigated a glenoid component with 3 cemented peripheral pegs and a central fluted peg that was supposed to provide fixation by bone ingrowth around the uncemented flanges of the central peg [21]. However, that study had many dropouts and high migration; 6 of 11 components had a MTPM greater than 1.5 mm at 6 months, which continued to increase, indicating a low grade of stability.

A study on an all-polyethylene pegged glenoid component had a mean MTPM of 0.98 mm in the non-erosion group, which is comparable to our findings. Rahme et al. investigated the stability of in-line pegged and keeled glenoid components and described their highest rotation to be less than –1.24° [31]. Streit et al. analyzed 9 keeled and 2 pegged components and found a mean rotation of 2.6° around the x-axis and 3.3° around the z-axis [32]. In our study, the highest mean rotation along the x-axis was –0.24° and the highest mean rotation was on the z-axes with –1.7° (valgus rotation), indicating that our study demonstrated the lowest mean rotation yet found.

Looking at the published results so far, the MTPM we found after 24 months is similar to or lower than that reported in previous studies of all-polyethylene components. Comparison between studies, however, should be made with caution, due to different RSA setups (different software, marker size, calibrations box, marker vs non-marker RSA). Further studies for comparison between glenoid designs would be of interest. More definitive conclusions may be supported by randomized controlled trials in a clinical setting, whereas registry studies would be appropriate for evaluating long-term implant survival.

Although the MTPM is calculated as a vector and is expected to be more sensitive to translation, substantial rotation about the z-axis may partly explain the micromovement (MTPM) observed within the first 6 months. A pronounced varus rotation of the glenoid component is a phenomenon that has also been documented in all-polyethylene glenoid designs [19,31]. Glenohumeral joint kinematics is characterized by translation of the humeral head in the glenoid socket during arm elevation: at the beginning of elevation in a superior direction, later during elevation in an inferior direction [33]. This leads to excentric loads on the glenoid, which has been suggested to create shear forces and valgus–varus rotation of the glenoid component [13]. Alternate rim-loading during movement of the arm with resulting rotational movement of the glenoid component has been described as the “rocking-horse phenomenon” and suggested a possible mechanism for loosening [5].

The clinical performance of hybrid components like the present ones has been investigated in larger single-cohort series with mid- to long-term follow-up [9–11]. In these studies, the revision rate due to aseptic loosening is reported as below 0.8%, which is in accordance with our study. No data from arthroplasty registers that specifically reports revision rates of hybrid glenoid was found.

### Strengths

A strength of this study is the relatively large number of cases included compared with previous studies.

### Limitations

The study had many dropouts, some due to patients missing the follow-up, but most related to technical difficulties in relation to the radiographic examination or analysis. In particular, missing matched markers around the glenoid was a challenge, which was often due to the humeral head overlying glenoid markers. Also, the glenoid component is relatively small, making it difficult to create a well-defined 3D body, resulting in unacceptably high condition numbers in some cases. These difficulties related to RSA of glenoid components have been recognized in other studies as well [13,16]. Using model-based RSA would overcome some of the problems related to markers, but this requires a well-defined metallic surface of the component that can be expressed as a 3D model, which was not possible in this study due to the porous surface of the central post. In addition, marker-based RSA is noted for higher accuracy and precision than model-based RSA [29,30]. As the dropouts in this study are mostly related to technical problems and some patients not showing up for all their radiographic controls, we believe it is less likely that the dropouts have introduced bias to the results. Also, considering the consecutive inclusion of patients, we find it reasonable to regard the analyzed cohort as a representative sample of patients with primary osteoarthritis.

### Conclusion

We found significant migration of the hybrid glenoid components during the first 24 months. Migration occurred mainly within the first 6 months and was dominated by valgus rotation. Thereafter the migration reached a plateau phase indicating that stability was achieved. Due to the few observations and the relatively short study period, association between early migration and later revision could not be evaluated.

*In perspective,* despite indications of solid fixation in our data, studies comparing hybrid with standard all-polyethylene components are needed to clarify potential differences in performance.

### Supplementary data

Tables 4–5 are available as Supplementary data on the article page, doi: 10.2340/17453674.2025.44953

## Supplementary Material



## Notes

AAC: conceptualization, data acquisition and analysis, drafting and reviewing; MT: data interpretation, drafting and reviewing; ML: data interpretation, reviewing; HCH; data analysis and interpretation, drafting and reviewing; SLJ: conceptualization, data acquisition and analysis, drafting and reviewing.

The authors would like to thank the RSAcore group, especially Bart Kaptein Dr. Ir., for his advice and help.

Handling co-editor: Li Felländer-Tsai

*Acta* thanks Hanna Cecilia Björnsson Hallgren, Antti P Launonen and Arne Lundberg for help with peer review of this manuscript.
