# Supplementary material for: Hybrid glenoid component migration after total shoulder arthroplasty: a cohort study using radiostereometric analysis with 2 years’ follow-up
Source: Acta Orthop. 2025 Nov 13;96:836–42. doi: 10.2340/17453674.2025.44953 (PMC12614002; doi:10.2340/17453674.2025.44953)
Supplement: Supplementary file 1 [file ActaO-96-44953-s1.pdf]

SUPPLEMENTARY TABLES

Table 4. RSA for revision cases CASE 1

	6 weeks	3 months	6 months	12 months
**Translation**				
X	0.08	–0.02	–0.46	–0.09
Y	0.26	0.14	0.09	0.05
Z	0.24	0.09	–0.19	–0.44
**Rotation**				
X	–0.41	0.22	–0.07	0.45
Y	–2.34	0.86	–0.39	–0.74
Z	0.33	0.09	6.98	8.23
**MTPM**	0.73	0.23	1.88	1.80

Table 5. RSA for revision cases CASE 2

	6 weeks	3 months	6 months	12 months
**Translation**				
X			–0.04	0.12
Y			0.04	0.01
Z			0.49	–0.08
**Rotation**				
X			–1.68	–0.01
Y			4.06	–1.17
Z			1.40	0.16
**MTPM**			1.27	0.41
